# Outcome of Stromal Vascular Fraction-Enriched Fat Grafting Compared to Intramuscular Transposition in Painful End-Neuromas of Superficial Radial Nerve: Preliminary Results

**DOI:** 10.3389/fsurg.2018.00010

**Published:** 2018-02-16

**Authors:** Simon Zimmermann, Richard M. Fakin, Pietro Giovanoli, Maurizio Calcagni

**Affiliations:** ^1^Division of Plastic and Hand Surgery, University Hospital Zurich, Zurich, Switzerland

**Keywords:** symptomatic neuroma, pain, stromal vascular fraction, fat grafting, SVF

## Abstract

**Introduction:**

The management of painful end-neuromas of the superficial branch of the radial nerve (SBRN) remains challenging due to high levels of pain relapse. The novel technique of stromal vascular fraction (SVF)-enriched fat grafting showed continuous pain relief, although failed to prove statistically significant. Besides acting as a mechanical barrier, SVF-enriched fat grafting might also affect the cellular level. The aim of this study was to compare clinical outcomes of SVF to the widely popular intramuscular transposition technique.

**Patients and methods:**

In this cohort study, 10 consecutive patients treated for painful end-neuromas of the SBRN between 2010 and 2013 were analyzed retrospectively. Microsurgical resection of end-neuromas was performed in all patients. Five patients were treated with subsequent intramuscular transposition into the brachioradialis muscle and five patients received SVF-enriched fat grafting. Five different pain modalities and various predictors were compared pre- and up to 36 months post-operatively.

**Results:**

In the transposition group, sustained pain reduction was not observed after an initial significant reduction 2 months’ post-surgery, resulting in pain relapse at 36 months and comparable to the preoperative assessment. In the graft group, some degree of pain reduction was observed at 2 months after the surgery and proved to be constant in the long-term outcome, although not statistically significant compared to preoperative levels.

**Conclusion:**

Both SVF-enriched fat grafting and intramuscular transposition failed to prove statistical significant pain reduction in treating symptomatic neuromas of peripheral nerves.

## Introduction

The management of painful end-neuromas of the superficial branch of the radial nerve (SBRN) remains difficult with multitude of existing proposed surgical treatments. Principally, neuroma excision and/or repositioning of the nerve stump into neighboring muscles (1, 2), bone (3), and veins (4) were proposed. Further attempts, such as stripping off the whole branch of the nerve (5), centro-centralization (6), or coverage with vascularized flaps (7) have been attempted. All of these techniques share the main concept of providing a mechanic barrier during nerve regeneration in order to reduce the messy axonal sprouting of terminal neuromas (8).

Recently, our group reported the novel treatment for symptomatic neuromas of the SBRN by stromal vascular fraction (SVF)-enriched fat grafting (9). SVF is a heterogeneous cell population, including concentrated amount of adipose-derived stem cells (ADSC) (10). SVF-enriched fat grafting might also affect the cellular level by a variety of biological mechanisms, including angiogenesis, differentiation potential, immunomodulatory properties, and secretion of extracellular matrix (11). The SVF is added to the fat graft in order to decrease the resorption rate and boost the regenerative potential of fat graft. Thus, the mechanical barrier provided by the fat graft might be maintained continuously during nerve regeneration, and SVF-enriched fat grafting might reduce the disorganized sprouting of terminal neuromas and subsequent nerve adherences.

Our small collective of five patients was the largest ever published series reporting SVF-enriched fat grafting in a single nerve population, although failed to show statistically significant pain reduction. However, a trend in continuous pain reduction could be demonstrated (9).

Indeed, none of described treatments resulted in superior outcome yet, and hence failed to establish a therapy consensus considering high rates of pain relapse (12). Furthermore, various prognostic factors for inferior pain relief following the surgery were described previously, including duration of pain for over 48 months, employment status, complex regional pain syndrome (CRPS) II symptoms, and smoking habits (12).

This cohort study compares a long-term outcome of SVF-enriched fat grafting as a novel surgical option for symptomatic end-neuromata of SBRN to the still frequently assessed alternative of intramuscular transposition of the nerve stump.

## Patients and Methods

This study was carried out in accordance with the recommendations of the University of Zurich Ethics Committee with written informed consent from all subjects. All subjects gave written informed consent in accordance with the Declaration of Helsinki. The protocol was approved by the University of Zurich Ethics Committee (BASEC-Nr. Req-2016-00655).

All patients operated on painful end-neuromata of the SBRN between 2010 and 2013 in our institution were analyzed retrospectively. From 2010 to 2012, five consecutive patients were treated with neuroma excision and intramuscular transposition into the brachioradialis muscle (the transposition group) and from 2012 to 2013 five consecutive patients received SVF-enriched fat grafting (the graft group). All the surgeries were performed by the same surgeon. Duration of pain, employment status, previously diagnosed CRPS II symptoms, and smoking habits of all patients were documented prior to the surgery. The data of both groups were statistically analyzed and compared. Symptoms of CRPS II were often primarily diagnosed in peripheral hospitals and not confirmed by specialists in our institution.

In order to confirm the diagnosis, all relevant nerve blocks were performed proximally to the neuroma in the middle third of the forearm in order to exclude pain from overlapping nerves, such as the lateral antebrachial cutaneous nerve (LACN) (13), posterior interosseous nerve (PIN), and a dorsal branch of ulnar nerve. In sequential order, all lidocaine blocks were completed by the same neurologist under sonographic control. The block for the SBRN was performed approximately 12–15 cm proximal to the lesion at the mid forearm. The LACN was blocked 10 cm distal to the biceps tendon on the forearm. Finally, the block for posterior interosseous nerve and for a dorsal branch of ulnar nerve was applied. Preoperative CRPS II and centralization of the pain were excluded analogously. Surgery was only performed in patients with considerable pain reduction [at least five VAS points on the Visual Analog Scale (VAS) for pain]. Pain modalities were evaluated as proposed by Elliot et al. (14). Spontaneous pain, spikes, hyperesthesia, tap pain, and motion pain affecting the skin over the neuroma were rated by our patients from 0 to 3 for each pain modality [none (0), mild (1), moderate (2), and severe pain (3)]. The clinical evaluation of pain was performed prior to surgery and during the follow-up at 2, 6, 12, and 36 months. Pain modalities were postoperatively evaluated both over the original site of painful neuroma, and over either the site of intramuscular transposition or of SVF-enriched fat grafting. In patients with persistent pain following the surgery, the diagnostic nerve blocks were repeated and the results documented.

### Surgical Procedure

#### Transposition Group

**Figure 1:**
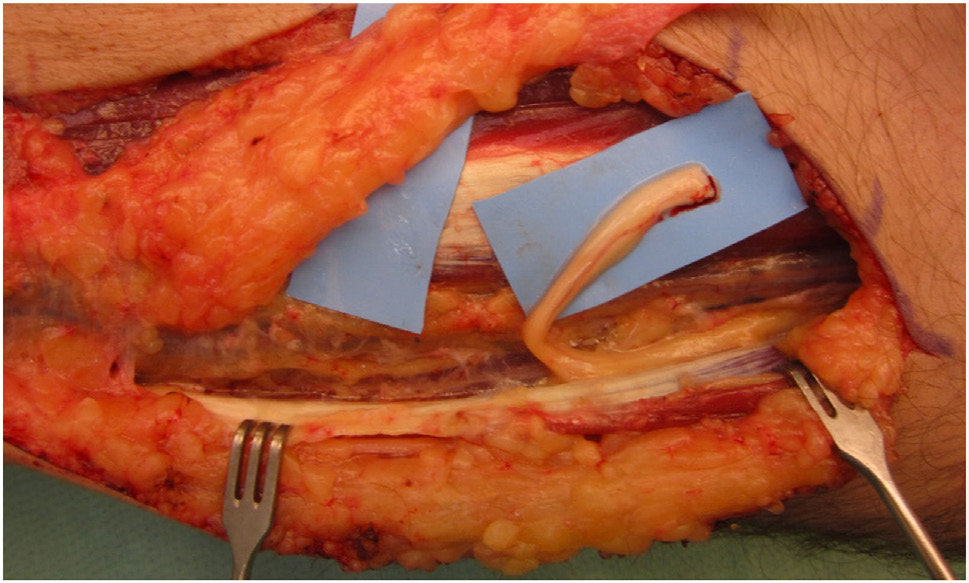
After neuroma removal, the remaining proximal nerve stump of the superficial branch of the radial nerve was prepared for transposition into the brachioradialis.

Under the microscope, the painful neuroma was resected and dissection of the proximal nerve stump was performed until the sufficient length was obtained for tension-free relocation into the brachioradialis muscle (Figure 1). A recipient site was created by a gentle and atraumatic division of the muscle fibers. The nerve stump was translocated 2 cm deep into the muscle. The nerve stump was consequently fixated with simple stitches with polypropylene 9–0 from the epineurium to the epimysium.

#### SVF-Enriched Fat Graft Group

**Figure 2:**
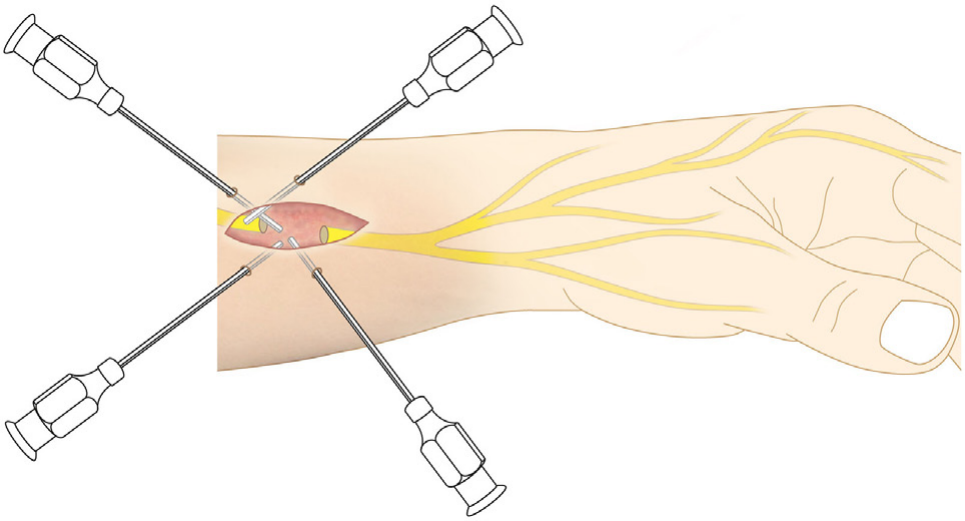
As application tool, four blunt cannulas were transcutaneously inserted and perineurally arranged around the nerve stump.

**Figure 3:**
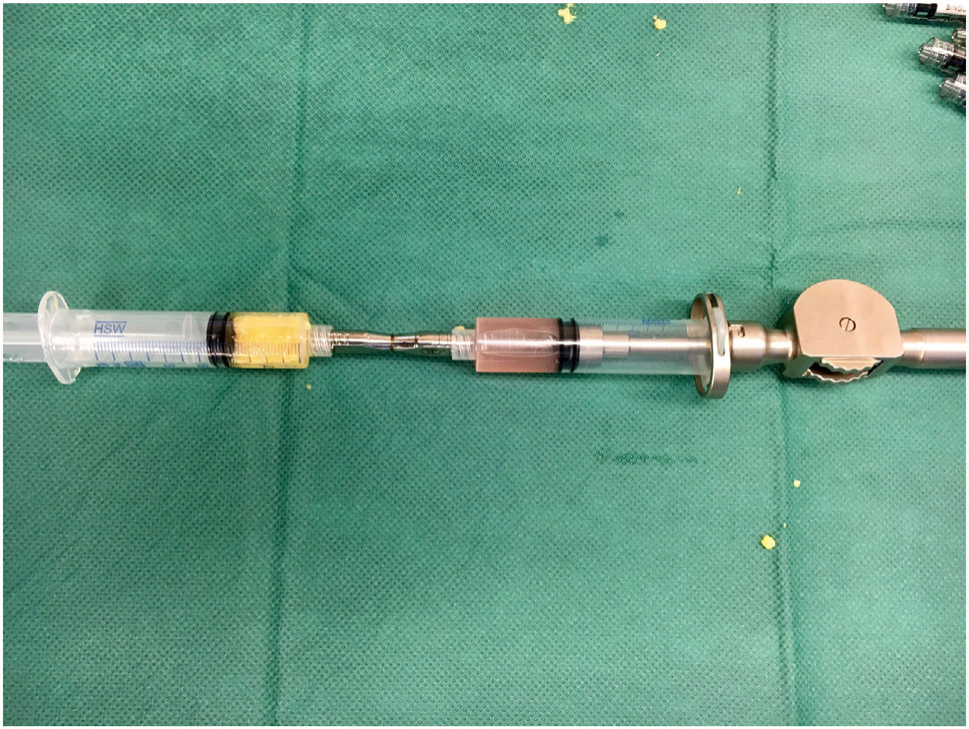
Communicating syringes were used to mix 5 ml of the concentrated stromal vascular fraction (SVF) with 2 ml of the sedimented lipid fraction in order to get the SVF-enriched fat graft.

A minimum of 220 ml of lipoaspirate was harvested through tumescent liposuction. In conformity with the manufacturers guidelines, the lipoaspirate was transferred into the Celution^®^ 800/CRS System and processed by one ampule of Celase^®^ processing enzyme reagent (Cytori Therapeutics, San Diego, CA, USA) (15). Meanwhile, the neuroma was excised under the microscope and subsequently neurolysis was performed. The nerve stump was dissected for at least 3 cm proximally to the wrist. Four small 1 mm transcutaneous incisions were arranged around the principal approach and blunt cannulas were placed perineurally to the nerve and secured in place by adhesive dressing (Figure 2). The wound was tightly closed with the blunt cannulas *in situ*. Then 5 ml of the processed SVF and 2 ml of the aspirated and sedimented lipid fraction were shuffled by communicating syringes (Figure 3). Finally, the SVF-enriched fat graft was injected perifocal to the nerve stump *via* the cannulas. The cannulas were removed and the incisions sutured.

### Statistical Analysis

The data were analyzed using the IBM Statistical Package for Social Sciences, version 22 (SPSS, Chicago, IL, USA) and presented as mean ± SDs. The Kolmogorov–Smirnov test was employed to verify the nonparametric nature of the study population and confirmed a nonexistent empirical distribution. Based on the nonparametric nature of the study population, the Wilcoxon’s test for related samples was applied to compare preoperative and postoperative results. The Mann–Whitney *U* test was used to verify statistical significance between the respective pain scores of each group. Statistical significance was considered at *p*-value less than 0.05.

## Results

The baseline characteristics of the study population are presented in Table 1. Eight male and two female patients with adequate pain reduction following the nerve blockade were included with a mean age of 41.5 ± 13.2 years. In eight patients, the neuromas of the SBRN manifested due to a previous trauma and in two patients due to an iatrogenic injury during orthopedic interventions. The mean duration of preoperative pain was 45.35 ± 46.98 months, ranging from 5 months to 12 years. Six patients had symptoms of CRPS II in their past medical history, which was documented in a peripheral hospital and could neither be confirmed by the surgeon nor by the neurologist practicing in our institution. Four patients were employed and six were unemployed. Nine patients smoked at the time of the surgical management (mean duration: 13.9 ± 10.7 pack years).

**Table 1:** Baseline characteristics.

	Overall	Transposition group	Graft group
Sex—male	8	3	5
—female	2	2	0

Injury—trauma	8	4	4
—iatrogenic	2	1	1

Mean age (in years)	41.5 ± 13.2	39.2 ± 8.79	49.8 ± 16.59

Mean duration of preoperative pain (in months)	45.35 ± 46.98	23.2 ± 16.48	67.5 ± 58.88

Complex regional pain syndrome II symptoms—Yes^a^	6	5	1
—No	4	0	4

Employment status—employed	4	2	2
—unemployed	6	3	3

Smoking—Yes (mean pack years)	9 (13.9 ± 10.7)	5 (8.55 ± 6.31)	4 (19.25 ± 9.43)
—No	1	0	1

*^a^CRPS II was documented in a peripheral hospital and could neither be confirmed by the surgeon nor by the neurologist practicing in our institution*.

The standard postoperative immobilization period amounted 4 weeks in the transposition group and 10 days in the SVF-enriched fat grafting group.

The comparison of pre- and postoperative pain scores of the two groups are listed in Tables 2–6 for each pain modality.

**Table 2:** Mean spontaneous pain ± SDs (*p*-value).

	Transposition group	Graft group
Preoperative score	2.2 ± 0.84	1.6 ± 0.55
2 months^a^	0.4 ± 0.55 (0.066)	0.8 ± 0.84 (0.102)
6 months^a^	1.8 ± 0.84 (0.157)	1.6 ± 1.52 (1.0)
12 months^a^	2 ± 0.71 (0.317)	1.6 ± 1.52 (1.0)
36 months^a^	1.4 ± 1.34 (0.157)	1.2 ± 1.1 (0.414)

*^a^Months after the surgery*.

*p-Value shows the difference between the pre- and postoperative scores*.

**Table 3:** Mean spikes ± SDs (*p*-value).

	Transposition group	Graft group
Preoperative score	2.6 ± 0.55	2.2 ± 1.3
2 months^a^	1 ± 0.71 (0.038)	1.4 ± 1.52 (0.285)
6 months^a^	2.2 ± 0.84 (0.157)	1.8 ± 1.64 (0.655)
12 months^a^	2.4 ± 0.89 (0.317)	1.8 ± 1.64 (0.655)
36 months^a^	2.6 ± 0.55 (1.0)	1.4 ± 1.34 (0.180)

*^a^Months after the surgery*.

*p-Value shows the difference between the pre- and postoperative scores*.

**Table 4:** Mean hyperaesthesia ± SDs (*p*-value).

	Transposition group	Graft group
Preoperative score	2.8 ± 0.45	1.6 ± 1.14
2 months^a^	1.4 ± 0.89 (0.038)	1.2 ± 1.64 (0.713)
6 months^a^	1.8 ± 1.3 (0.102)	1.4 ± 1.52 (0.713)
12 months^a^	2.2 ± 1.1 (0.180)	1.4 ± 1.52 (0.713)
36 months^a^	2.2 ± 0.84 (0.180)	1.2 ± 1.64 (0.713)

*^a^Months after the surgery*.

*p-Value shows the difference between the pre- and postoperative scores*.

**Table 5:** Mean tap pain ± SDs (*p*-value).

	Transposition group	Graft group
Preoperative score	3 ± 0.0	2.8 ± 0.45
2 months^a^	0.8 ± 0.84 (0.041)	1.6 ± 0.89 (0.083)
6 months^a^	2.6 ± 0.55 (0.157)	1.6 ± 1.52 (0.157)
12 months^a^	3 ± 0.0 (1.0)	1.8 ± 1.3 (0.180)
36 months^a^	2.8 ± 0.45 (0.317)	1.8 ± 1.3 (0.197)

*^a^Months after the surgery*.

*p-Value shows the difference between the pre- and postoperative scores*.

**Table 6:** Mean motion pain ± SDs (*p*-value).

	Transposition group	Graft group
Preoperative score	2.8 ± 0.45	2.8 ± 0.45
2 months^a^	0.2 ± 0.45 (0.034)	1.4 ± 1.52 (0.102)
6 months^a^	2 ± 0.71 (0.102)	1.2 ± 1.64 (0.102)
12 months^a^	2.4 ± 0.55 (0.157)	1.6 ± 1.52 (0.109)
36 months^a^	1.6 ± 1.52 (0.194)	1.4 ± 1.34 (0.066)

*^a^Months after the surgery*.

*p-Value shows the difference between the pre- and postoperative scores*.

**Figure 4:**
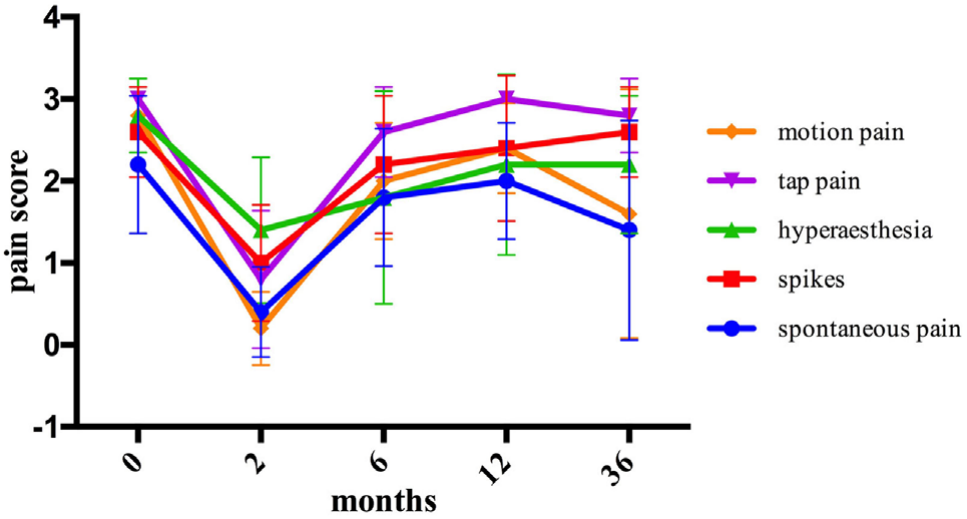
The assessment of pain modalities before and after intramuscular transposition.

Statistically significant pain reduction could be shown in the transposition group 2 months after the surgery for the majority of pain modalities and on overall pain assessment. Afterward, subsequent increase of pain was detected and failed to maintain statistical significance from 6 months’ post-surgery onward. An overall pain reduction from 36 months after the surgery was assessed as follows: spontaneous pain from 2.2 ± 0.84 to 1.4 ± 1.34 (*p* = 0.157), hyperesthesia from 2.8 ± 0.45 to 2.2 ± 0.84 (*p* = 0.180), tap pain from 3 ± 0.0 to 2.8 ± 0.45 (*p* = 0.317), and motion pain from 2.8 ± 0.45 to 1.6 ± 1.52 (*p* = 0.194). Spikes were assessed 36 months after the surgery at the same level as before the surgery (*p* = 1.0) (Figure 4).

**Figure 5:**
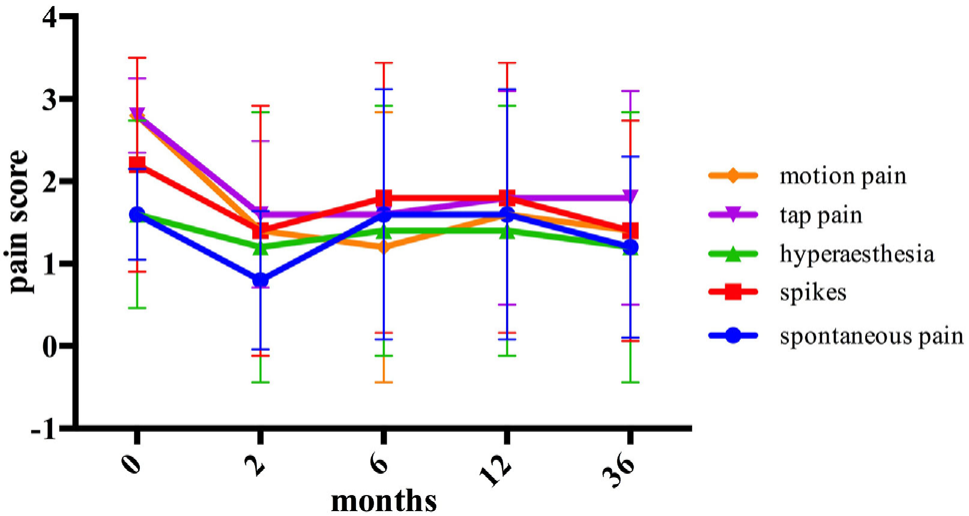
The assessment of pain modalities before and after stromal vascular fraction-enriched fat grafting.

The graft group showed a relevant reduction in all pain modalities 2 months after the surgery, although the difference did not reach statistical significance. However, pain reduction appeared to be maintained during 36 months after the surgery. Spontaneous pain could be reduced from 1.6 ± 0.55 to 1.2 ± 1.1 (*p* = 0.414), spikes from 2.2 ± 1.3 to 1.4 ± 1.34 (*p* = 0.180), hyperesthesia from 1.6 ± 1.14 to 1.2 ± 1.64 (*p* = 0.713), tap pain from 2.8 ± 0.45 to 1.8 ± 1.3 (*p* = 0.197), and motion pain from 2.8 ± 0.45 to 1.4 ± 1.34 (*p* = 0.066) (Figure 5).

**Figure 6:**
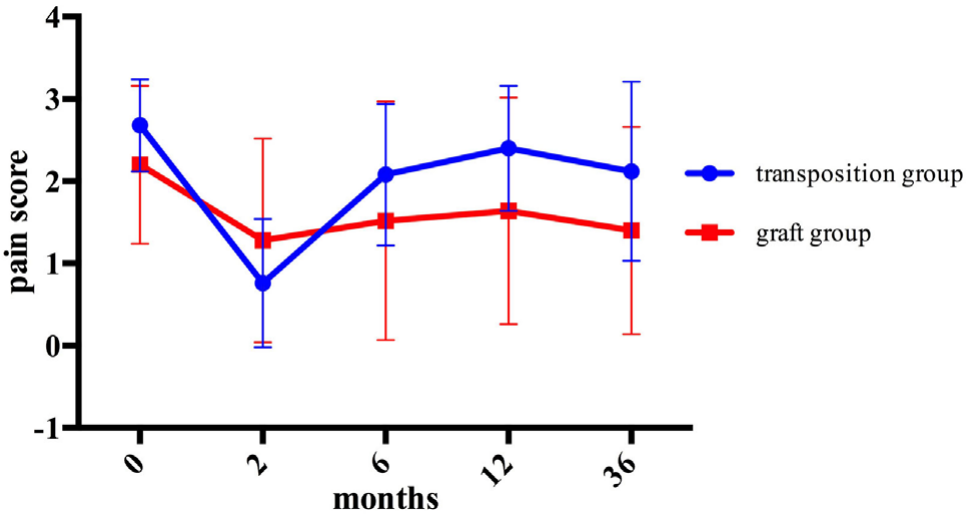
Comparison of overall assessment of pain modalities for each group.

The overall assessment of pain modalities in each group is shown and compared in Figure 6. Comparing the mean pre- and postoperative pain scores after 2, 6, 12, and 36 months of the two groups, no statistical significance was detected.

## Discussion

The aim of this cohort study was to compare the long-term outcome of SVF-enriched fat grafting to the widely popular intramuscular transposition in the treatment of painful end-neuromas of the SBRN. Our data suggest no significant pain reduction by SVF-enriched fat grafting compared to intramuscular transposition in neuromal pain over an observation period of 3 years. However, SVF-enriched fat grafting shall provide a beneficial action on cellular level by a variety of biological mechanisms, including angiogenesis, differentiation potential, immunomodulatory properties, and secretion of extracellular matrix (11).

Neuromas of the SBRN are associated with a surgical success rate of only 33% (12). The surgical procedure is often performed after a long regime of non-operative pain management. Vaienti et al. reported a series of promising results of perineurial fat grafting (PFG) without the SVF in eight patients suffering of painful neuromas of the upper limb (16, 17). However, pain reduction failed to show a statistical significance from 6 months postoperatively onward with pain relapse 12 months after the PFG (16, 17).

In previous trials, ADSCs-enriched adipose grafts showed a considerably reduced absorption rate in comparison to non-enriched fat grafts (18). ADSC as part of the SVF activate angiogenesis and the expression of growth factors, such as VEGF and bFGF on cellular level (19). Thus, acting as a mechanic barrier, the SVF-enriched fat graft might be sustained continuously, particularly in the vulnerable period of nerve regeneration.

Besides its impact as a mechanical layer, the therapeutic effect of SVF-enriched fat grafting is believed to be based on a cellular level. And therefore, proving a potential answer to the proposed partial chemical component in neuromal pain (20, 21). An intact perineurium is believed to be of utmost importance in neuromal formation. Undamaged perineurium presents an impermeable membrane in nerve regeneration and prevents disorganized terminal sprouting of nerve fibers (22). During axonal outgrowth in nerve regeneration, Schwann cells play a dominant role (23). SVF-enriched nerve conduits were shown to be responsible for an accelerated axon regeneration and a significantly higher number and diameter of myelinated fibers in a test series on sciatic nerves in rats (24). Interestingly, the application of undifferentiated ADSCs in an artificial nerve conduit in a rat sciatic nerve model, resulted in a detection of only a small amount of viable ADSCs 2 weeks after the transplantation (25). Hence, rather than playing a dominate role in direct neuronal differentiation, ADSCs might contribute to nerve regeneration through initially boosting neurotrophic factors, such as BDNF, NGF, and GDNF and their interactivity with SC (25, 26). Concluding, SVF-enriched fat grafting might present a relevant component in nerve regeneration, particularly in providing sufficient myelinated fibers for an organized nerve outgrowth, and potential prevention of the messy axonal sprouting of terminal neuromas (9).

In addition, SVF-enriched fat grafting might provide biologic mechanisms, such as immunomodulation and secretion of extracellular matrix as local inflammation and following immune response are crucial parts in the process of nerve regeneration (11). Immunomodulation is observed by hematopoietic cells, such as macrophages of the M2 phenotype or T helper cells and natural killer cells in the SVF which provide anti-inflammatory function by secreting of numerous cytokines (27). Furthermore, the cellular signaling is influenced, though a decrease of pro-inflammatory cytokines like TNF-α and IL-6 (28). Cellular signaling and migration is influenced *via* the expression of matrix components by fibroblasts in the SVF (11).

In our results, some degree of improvement was manifested following SVF-enriched fat grafting, though pain reduction did not prove to be statistically significant. However, pain reduction was shown continuous at a steady state from 2 months postoperatively onward, with no pain relapse up to at least 36 months. Consequently, this favorable trend might be associated both with a greater long-term residual volume of the fat graft due and the proposed biologic mechanisms attributable to the SVF (11, 19). Concluding, our preliminary results in pain reduction are in accordance with other results published in the field of autologous fat injections in painful neuromas (29).

The processed SVF should be added to the fat graft in the proposed optimum of 10,000 cells per 200 µl adipose tissue as higher amounts of SVF significantly increase the resorption rate (30). Moreover, the regenerative potential of the fat graft is decreased with higher fractions.

Despite centralization of pain and preoperative CRPS II were excluded by preoperative nerve blocks, the chance of success in pain reduction is *a priori* inferior after long periods of unsuccessful pain therapy. Furthermore, the anatomical overlap between SBRN and LACN might be an underestimated factor for decreased pain relief. Although verifying preoperative nerve blocking was performed to confirm the diagnosis, postoperative disorganized outgrowth of the LACN in the denervated common cutaneous innervation area cannot be excluded. In patients with persistent pain post-surgery, the diagnostic nerve blocks, especially of the LACN, were repeated. If considerable pain reduction could be shown, surgical follow-up intervention was offered to the patients. Resection of the LACN was secondarily performed in one patient in the transposition group following an 18 months regime of non-operative pain management. This additional surgical intervention, however, resulted in only minor pain improvement. Two patients in the graft group with minor or no improvement of pain received repeated diagnostic nerve blocks, with a special attribute to the LACN. Nonetheless, considerable pain reduction could not be achieved. Patients with persisting pain post-surgery were continuously followed in our specialized pain unit.

Prognostic risk factors for inferior pain relief were described previously (12). Most of our patients were unemployed with current smoking habits at the time of the surgery and had documented CRPS II symptoms in their past medical history. The mean duration of preoperative pain was 67.5 months in the graft group, representing a further unfavorable predictor as compared to the transposition group. Regardless of which operative technique was assessed, insufficient pain reduction was observed in patients with at least two out of four proposed predictors. Consequently, our results can confirm the negative influence of these proposed risk factors.

Both surgical techniques aim to protect the nerve stump against mechanical irritation, either by muscle or fat graft, in order to prevent persistent and disorganized axonal outgrowth. Pain reduction in SVF-enriched fat grafting failed to show a statistical significance as compared to the muscular transposition approach.

In the transposition group, sustained pain relief was not observed after an initial significant symptom reduction at 2 months after the operation, resulting in pain 36 months’ post-surgery comparable to the preoperative status. Notably, this pattern was not observed in the graft group. In contrast, the long-term outcome and particularly pain relief appeared to be sustained from 2 months postoperatively onward.

The initial significant pain reduction achieved in the transposition group is likely due to neuroma resection itself. However, with subsequent continuous axonal outgrowth, the muscle does not appear to offer the desired protective effect during the nerve regeneration. In contrast, we speculate that the continuous pain reduction in the graft group is probably due to persistent biological effects of the SVF indeed.

Besides, SVF-enriched fat grafting does not require the surgical intervention of the brachioradialis muscle and offers a much shorter immobilization period of the wrist. This needs to be taken into consideration when evaluating the more expensive and time-consuming operative intervention by SVF-enriched fat grafting.

This cohort study is limited both due to the retrospective design and small number of patients. Additionally, a slight difference was noticed in the preoperative pain score between the fat grafting group and transposition group. As neuromas of the SBRN are a rare condition and their surgical approach even less common, all patients operated on neuromas of the SBRN between 2012 and 2013 either by fat grafting or intramuscular transposition were included, independently of their preoperative pain score. Hence, the difference in pain reduction within both groups was compared instead of pre- and postoperative pain scores.

Although the present series is the largest to investigate the outcome of SVF-enriched fat grafting on a single nerve population up to date, future randomized studies with more patients are necessary in order to validate the assessment of this novel treatment (21).

## Conclusion

Stromal vascular fraction-enriched fat grafting is a novel approach for the complex treatment of painful end-neuromas of the SBRN. In this study, however, both SVF-enriched fat grafting and intramuscular transposition failed to prove statistical significant pain reduction in treating of symptomatic neuromas of peripheral nerves. As SVF-enriched fat grafting yields promising results in laboratory setting, clinical investigations of the exact mechanisms of action, and subsequent validation in larger clinical trials are needed.

## Ethics Statement

This study was carried out in accordance with the recommendations of the University of Zurich Ethics Committee with written informed consent from all subjects. All subjects gave written informed consent in accordance with the Declaration of Helsinki. The protocol was approved by the University of Zurich Ethics Committee (BASEC-Nr. Req-2016-00655).

## Author Contributions

All authors (SZ, RF, MC, and PG) are substantial contributors to the conception of the manuscript, took part in revising the content of the work critically, and gave their final approval of the current version to be published. Questions related to the accuracy or integrity of any part of the work are appropriately investigated and resolved by all authors (SZ, RF, MC, and PG).

## Conflict of Interest Statement

The authors declare that the research was conducted in the absence of any commercial or financial relationships that could be construed as a potential conflict of interest.

## References

[1] Mackinnon SE Dellon AL. Results of treatment of recurrent dorsoradial wrist neuromas. Ann Plast Surg (1987) 19(1):54–61. 10.1097/00000637-198707000-00009 3631861

[2] Hazari A Elliot D. Treatment of end-neuromas, neuromas-in-continuity and scarred nerves of the digits by proximal relocation. J Hand Surg Br (2004) 29(4):338–50. 10.1016/j.jhsb.2004.01.005 15234497

[3] Goldstein SA Sturim HS. Intraosseous nerve transposition for treatment of painful neuromas. J Hand Surg Am (1985) 10(2):270–4. 10.1016/S0363-5023(85)80120-9 3980943

[4] Koch H Haas F Hubmer M Rappl T Scharnagl E. Treatment of painful neuroma by resection and nerve stump transplantation into a vein. Ann Plast Surg (2003) 51(1):45–50. 10.1097/01.SAP.0000054187.72439.57 12838124

[5] Lanzetta M Nolli R. Nerve stripping: new treatment for neuromas of the palmar cutaneous branch of the median nerve. J Hand Surg Br (2000) 25(2):151–3. 10.1054/jhsb.1999.0355 11062572

[6] Barbera J Albert-Pamplo R. Centrocentral anastomosis of the proximal nerve stump in the treatment of painful amputation neuromas of major nerves. J Neurosurg (1993) 79(3):331–4. 10.3171/jns.1993.79.3.0331 8360727

[7] Adani R Tarallo L Battiston B Marcoccio I. Management of neuromas in continuity of the median nerve with the pronator quadratus muscle flap. Ann Plast Surg (2002) 48(1):35–40. 10.1097/00000637-200201000-00005 11773728

[8] Lutz BS Ma SF Chuang DC Chan KH Wei FC. Interposition of a pedicle fat flap significantly improves specificity of reinnervation and motor recovery after repair of transected nerves in adjacency in rats. Plast Reconstr Surg (2001) 107(1):116–23. 10.1097/00006534-200101000-00017 11176609

[9] Calcagni M Zimmermann S Scaglioni MF Giesen T Giovanoli P Fakin RM. The novel treatment of SVF-enriched fat grafting for painful end-neuromas of superficial radial nerve. Microsurgery (2016). 10.1002/micr.30122 27731522

[10] Zuk PA Zhu M Mizuno H Huang J Futrell JW Katz AJ Multilineage cells from human adipose tissue: implications for cell-based therapies. Tissue Eng (2001) 7(2):211–28. 10.1089/107632701300062859 11304456

[11] Guo J Nguyen A Banyard DA Fadavi D Toranto JD Wirth GA Stromal vascular fraction: a regenerative reality? Part 2: mechanisms of regenerative action. J Plast Reconstr Aesthet Surg (2015) 69(2):180–8. 10.1016/j.bjps.2015.10.014 26546112

[12] Stokvis A van der Avoort DJ van Neck JW Hovius SE Coert JH. Surgical management of neuroma pain: a prospective follow-up study. Pain (2010) 151(3):862–9. 10.1016/j.pain.2010.09.032 20974520

[13] Mackinnon SE Dellon AL. The overlap pattern of the lateral antebrachial cutaneous nerve and the superficial branch of the radial nerve. J Hand Surg Am (1985) 10(4):522–6. 10.1016/S0363-5023(85)80076-9 4020063

[14] Elliot D Sierakowski A. The surgical management of painful nerves of the upper limb: a unit perspective. J Hand Surg Eur Vol (2011) 36(9):760–70. 10.1177/1753193411423140 22058230

[15] Fraser JK Hicok KC Shanahan R Zhu M Miller S Arm DM The celution system: automated processing of adipose-derived regenerative cells in a functionally closed system. Adv Wound Care (New Rochelle) (2014) 3(1):38–45. 10.1089/wound.2012.0408 24761343 PMC3900001

[16] Vaienti L Merle M Battiston B Villani F Gazzola R. Perineural fat grafting in the treatment of painful end-neuromas of the upper limb: a pilot study. J Hand Surg Eur Vol (2013) 38(1):36–42. 10.1177/1753193412441122 22415427

[17] Vaienti L Gazzola R Villani F Parodi PC. Perineural fat grafting in the treatment of painful neuromas. Tech Hand Up Extrem Surg (2012) 16(1):52–5. 10.1097/BTH.0b013e31823cd218 22411121

[18] Kolle SF Fischer-Nielsen A Mathiasen AB Elberg JJ Oliveri RS Glovinski PV Enrichment of autologous fat grafts with ex-vivo expanded adipose tissue-derived stem cells for graft survival: a randomised placebo-controlled trial. Lancet (2013) 382(9898):1113–20. 10.1016/S0140-6736(13)61410-5 24075051

[19] Zhu M Dong Z Gao J Liao Y Xue J Yuan Y Adipocyte regeneration after free fat transplantation: promotion by stromal vascular fraction cells. Cell Transplant (2015) 24(1):49–62. 10.3727/096368913X675133 24172865

[20] Anand P Nerve growth factor regulates nociception in human health and disease. Br J Anaesth (1995) 75(2):201–8. 10.1093/bja/75.2.201 7577254

[21] Elliot D Commentary on Vaienti et al. Perineural fat grafting in the treatment of painful end-neuromas of the upper limb. J Hand Surg Eur Vol (2013) 38(1):43 10.1177/1753193412466203 23255179

[22] Yuksel F Kislaoglu E Durak N Ucar C Karacaoglu E. Prevention of painful neuromas by epineural ligatures, flaps and grafts. Br J Plast Surg (1997) 50(3):182–5. 10.1016/S0007-1226(97)91367-9 9176005

[23] Widgerow AD Salibian AA Lalezari S Evans GR. Neuromodulatory nerve regeneration: adipose tissue-derived stem cells and neurotrophic mediation in peripheral nerve regeneration. J Neurosci Res (2013) 91(12):1517–24. 10.1002/jnr.23284 24105674 PMC7061900

[24] Mohammadi R Sanaei N Ahsan S Rostami H Abbasipour-Dalivand S Amini K. Repair of nerve defect with chitosan graft supplemented by uncultured characterized stromal vascular fraction in streptozotocin induced diabetic rats. Int J Surg (2014) 12(5):33–40. 10.1016/j.ijsu.2013.10.018 24239939

[25] Erba P Mantovani C Kalbermatten DF Pierer G Terenghi G Kingham PJ. Regeneration potential and survival of transplanted undifferentiated adipose tissue-derived stem cells in peripheral nerve conduits. J Plast Reconstr Aesthet Surg (2010) 63(12):e811–7. 10.1016/j.bjps.2010.08.013 20851070

[26] Liu G Cheng Y Guo S Feng Y Li Q Jia H Transplantation of adipose-derived stem cells for peripheral nerve repair. Int J Mol Med (2011) 28(4):565–72. 10.3892/ijmm.2011.725 21687931

[27] Eto H Ishimine H Kinoshita K Watanabe-Susaki K Kato H Doi K Characterization of human adipose tissue-resident hematopoietic cell populations reveals a novel macrophage subpopulation with CD34 expression and mesenchymal multipotency. Stem Cells Dev (2013) 22(6):985–97. 10.1089/scd.2012.0442 23137270 PMC3585481

[28] Premaratne GU Ma LP Fujita M Lin X Bollano E Fu M. Stromal vascular fraction transplantation as an alternative therapy for ischemic heart failure: anti-inflammatory role. J Cardiothorac Surg (2011) 6:43. 10.1186/1749-8090-6-43 21453457 PMC3079611

[29] Baptista C Iniesta A Nguyen P Legre R Gay AM. [Autologous fat grafting in the surgical management of painful scar: preliminary results]. Chir Main (2013) 32(5):329–34. 10.1016/j.main.2013.07.006 24035685

[30] Paik KJ Zielins ER Atashroo DA Maan ZN Duscher D Luan A Studies in fat grafting: part V. Cell-assisted lipotransfer to enhance fat graft retention is dose dependent. Plast Reconstr Surg (2015) 136(1):67–75. 10.1097/PRS.0000000000001367 25829158 PMC4483157

